# A bioinspired optoelectronically engineered artificial neurorobotics device with sensorimotor functionalities

**DOI:** 10.1038/s41467-019-11823-4

**Published:** 2019-08-27

**Authors:** Mohammad Karbalaei Akbari, Serge Zhuiykov

**Affiliations:** 1 Centre for Environmental & Energy Research, Ghent University, Global Campus, Incheon, South Korea; 2 Department of Green Chemistry and Technology, Faculty of Bioscience Engineering, Ghent University, 9000 Ghent, Belgium

**Keywords:** Nanoscale materials, Nanobiotechnology, Bionanoelectronics

## Abstract

Development of the next generation of bio- and nano-electronics is inseparably connected to the innovative concept of emulation and reproduction of biological sensorimotor systems and artificial neurobotics. Here, we report for the first time principally new artificial bioinspired optoelectronic sensorimotor system for the controlable immitation of opto-genetically engineered neurons in the biological motor system. The device is based on inorganic optical synapse (In-doped TiO_2_ nanofilm) assembled into a liquid metal (galinstan) actuator. The optoelectronic synapse generates polarised excitatory and inhibitory postsynaptic potentials to trigger the liquid metal droplet to vibrate and then mimic the expansion and contraction of biological fibre muscle. The low-energy consumption and precise modulation of electrical and mechanical outputs are the distinguished characteristics of fabricated sensorimotor system. This work is the underlying significant step towards the development of next generation of low-energy the internet of things for bioinspired neurorobotic and bioelectronic system.

## Abstract (web summary)

The internet of things technologies relies on the development of sensorimotor systems. Here, Karbalaei Akbari and Zhuiykov show a bioinspired sensorimotor system based on an integration of an artificial optical synapse and a liquid metal actuator, which mimics the expansion and contraction of biological muscles.

## Introduction

An intricately interconnected biosensor network, neural signal processors and bio-motors enable the sustainable performance of human body^1,2^. The sensing functionalities including both external exciters and bioelectrical stimuli are detected by biosensors. The bilateral signal transmissions between the biosensors and decision making unit are facilitated by the nervous system^3,4^. Indeed, the biological synapses, as one of the key members of neural system, are the information channels ensuring short-term computation, long-term learning and memorisation by tuning the synaptic weights^5^. Uniqueness of the brain as the main intelligent organ arises from its highly energy efficient data processing. These functionalities are facilitated and executed by the network of synapses in the neural system^6^. In the human brain, the obtained information is processed and then orders are delivered to the corresponding autonomic and somatic nervous systems^7^. The combination of these interconnected events concludes one of the most complicated biological cycle, i.e., voluntary and involuntary movements^8^. To imitate the nervous system behaviour, it is vital to design an artificial electronic synapse, which receives the excitatory signals and then generates informative synaptic responses to the motor system. Thus, this imitation can fundamentally broaden the horizon of artificial neurorobotics and learning systems.

In general, optical cognition is predominantly based on the visible light-driven sensory mechanisms. The visual-based data processing accompanied by the auditory system facilitate perception, learning and understanding processes^9,10^. While the concept of opto-genetically engineered neuron in the biological motor system is now well recognised^11,12^, it has been recently announced that the optical stimuli can be employed to excite artificial synaptic devices^13^. Novel synaptic devices have efficiently responded to the optical excitation^13^. However, up to date the researchers’ attentions have mostly been focused on the development of materials and devices to imitate the synaptic behaviour in the human brain^14^. There are just a few cases that make a substantial step forward to confront the fundamental challenges of the development of biological sensorimotor systems^14,15^. In bioscience, an integration of the visible light-driven artificial synaptic device with a motor system can trigger several fundamental applications in cutting-edge technologies, including optical wireless devices^16^, light-driven robotics^17^, neurological optoelectronic sensorimotors^14^, microfluidic chips and nano-pumps in drug delivery systems^18^.

Principally new approach is proposed and executed in the present research. It consists of an artificial synapse innovatively incorporated into the liquid metal actuator device to imitate sensorimotor functions. In this device, the indium (In)-doped TiO_2_ optical synapse plays the role of visible light sensor, which receives optical signals and then generates informative postsynaptic current and potential pulses. The transfer of controlled potential pulses to the liquid metal actuator induces the mechanical motion, which in turn mimics the muscular contraction/relaxation in the artificial neuro-robotic device. The following investigation of mechanisms behind the mechanical motion provides valuable insights towards the motor function of fabricated sensorimotor system. This achievement is fulfilled by precise design and optimisation of sensorimotor components. Therefore, the bioinspired optoelectronic device and its innovative insights would undoubtedly open up new horizons for the next step in development of the different artificial sensorimotor systems and the internet of things technologies.

## Results

### Fundamental of artificial neuromuscular system

**Fig. 1:**
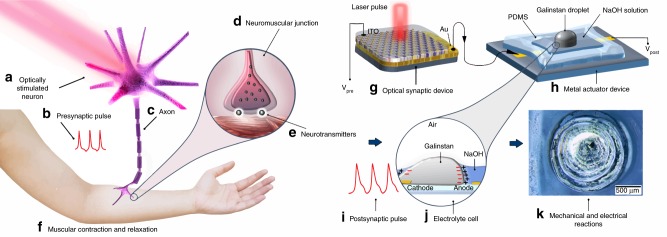
The scheme of opto-genetically engineered neuron system and artificial optoelectronic sensorimotor device. **a**–**f** In a biological system **a** the optically stimulated neurons, which contain photosensitive proteins generate **b** presynaptic pulses through **c** axons to **d** neuromuscular junctions. **e** Neurotransmitters convey the presynaptic pulses to muscle fibre to cause the **f** postsynaptic reactions like muscular contraction and relaxation. Analogously, **g**﻿–**k** in the artificial sensorimotor system **g** an optical synaptic device is integrated into a **h** metal actuator device. The optical synaptic device is triggered by pulsed lights to generate **i** postsynaptic pulses. The transferred postsynaptic potential pulses to the liquid metal actuator device oscillate the galinstan droplet in NaOH solution, which is in fact an **j** electrolyte cell. The outputs of device can be recorded either as **k** mechanical or electrical reactions

Optical control of muscle functions is facilitated by motoneurons in biological system. Photosensitive motoneurons are the genetically modified neurons that indirectly or directly innervate effector targets, which mainly control contraction of muscle fibres in neuromuscular junctions^19^. An optically stimulated neuron (Fig. 1a) generates and transfers presynaptic pulses (Fig. 1b) through axon (Fig. 1c). A neuromuscular junction (Fig. 1d) is a biological chemical synapse that transmits presynaptic action potentials to the muscle fibre by release of biological neurotransmitters in neuromuscular junctions (Fig. 1e). Following the delivery of neurotransmitters, excitatory postsynaptic potentials are generated in the muscle fibre resulting in muscle contraction^19^ (Fig. 1f). To imitate the biological motoneurons behaviour, a visible light-sensitive TiO_2_ optical synaptic device (Fig. 1g) is integrated into a liquid metal actuator (Fig. 1h), which acts as artificial muscular component. The employed doping technique has broadened the optical sensitivity of ultra-thin high-bandgap TiO_2_ film to visible light region. The reasons why a visible light-sensitive optical synapse is used return to its merit characteristics, including the resemblance to an optically stimulated synaptic system in motoneurons and also to the capability of output control by modulation of the conductance states. In our optical synapse, the control of the resistance of device by employing patterned light pulses with various intensities and frequencies enabled us to adjust the output of synaptic device, whereas the reaction to light stimulus is much faster and uncontrollable in the regular photosensors^20^. In doing so, developed optically stimulated synaptic device generates the output potentials transferrable to the liquid metal actuator (galinstan). The capability of the output control of optical device is critically important when the possibility of integration of artificial optical synapse with actuator as mechanical microcontrollers is concerned. The liquid metal droplet in the bath of NaOH solution technically constitutes an electrochemical cell (EC), which receives the postsynaptic pulses (Fig. 1i) from the optical device. The imposition of postsynaptic pulses to EC (Fig. 1j) leads to the reconfiguration of the charge distribution on the surface of galinstan droplet in NaOH solution^21^. It facilitates the mechanical oscillation of liquid metal in NaOH bath (Fig. 1k) resembling a neuromuscular electronic system in robotic devices. By applying patterned optical pulses, the weight and rhythm of the potential signals can be designed, and consequently, the motion of galinstan actuator can be controlled. The scheme of sensorimotor is presented in Supplementary Fig. 1 and Supplementary Note 1.

### Structural characteristics of In-doped TiO_2_ film

**Fig. 2:**
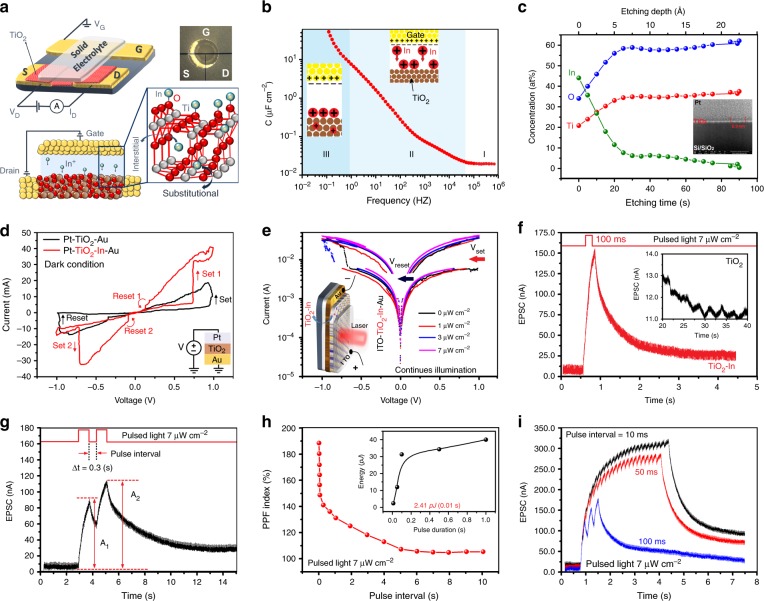
The characteristics of metal-oxide memristor and optical synaptic device. **a** The scheme of ion intercalation process in TiO_2_ film. **b** The capacitance-frequency *(c-f)* graph for In-ion intercalation process. **c** The XPS surface profile of atomic concentration. **d** The *I-V* sweep curves of Pt/TiO_2_/Au and Pt/In-doped TiO_2_/Au memristors under dark and **e** logarithmic scale *I-V* curves of Pt/In-doped TiO_2_/Au device under continues illumination of visible lights (530 nm) with various power densities. **f** The EPSC of TiO_2_ and In-doped TiO_2_ synaptic devices induced by 7 μW cm^−2^ laser pulses (530 nm) with 100 ms duration. **g** The EPSC of In-doped TiO_2_ synaptic devices induced by two successive 7 μW cm^−2^ laser pulses with 0.3 s intervals. **h** The variation of PPF index vs. pulse intervals for In-doped TiO_2_ synaptic devices. The inset shows the variation of energy of synaptic events per a singular optical pulse (7 μW cm^−2^) vs. pulse durations. **i** EPSC of In-doped TiO_2_ synaptic devices stimulated by pulsed lights with various pulse intervals

Nanostructured metal oxides are one of the main candidates for employment in synaptic devices since they benefit from the advantages of resistive switching, low-energy consumption and facile integration in complementary metal-oxide semiconductor (CMOS) technology^22^. Herein, the uniqueness of atomic layer deposition (ALD) technique facilitated the conformal deposition of ultra-thin 7 nm thick TiO_2_ film over the Au electrodes (Fig. 2a). The additional information is described in Supplementary Fig. 2 and Supplementary Note 2. Solid electrolyte (SE) containing Indium-ions (In-ions) was drop cast over source-drain and gate electrodes to cover TiO_2_ films and Au electrodes. To intercalate into TiO_2_ film, In-ions should, respectively, overcome the surface diffusion and bulk diffusion energy barriers^23^. By imposing a sufficient *V*_*G*_, the ions have higher chance to intercalate into TiO_2_ sub-layer. A direct (*dc*) gate voltage (*V*_*G*_) was applied on the SE/TiO_2_ interface to investigate the ion intercalation and then the corresponding *I*_DS_ was monitored at constant *V*_*D*_ = 100 mV (Supplementary Fig. 3 and Supplementary Note 3). The appearance of a large hysteresis by applying *dc* sweeping voltage (Supplementary Fig. 3) is an indication of the potential-induced ion intercalation into the oxide film^23^. Up on following increase of *V*_*G*_, In-ions were initially accumulated at the interface of SE/TiO_2_ film and then diffused into TiO_2_ sub-layers, which is manifestation of electrochemical doping^24^. The capacitance-frequency (*c-f*) measurement on Au/SE/TiO_2_ clarifies the dynamic nature of In-ion intercalation in TiO_2_ film. The capacitance variation vs. frequency enhancement (Fig. 2b) demonstrates the occurrence of three individual steps. At high-frequency region ( >10^5^ Hz) the value of specific capacitance is insignificant (≈0.020 μF cm^−2^), which refers to the capacitance of bulk electrolyte (Region ɪ). Further frequency decline is accompanied by the formation of electrolyte double layer (EDL) at SE/TiO_2_ interface^23^. The constant and rapid increase of capacitance following by the frequency decrement in region ɪɪ is manifestation of the ion migration and accumulation at the interface^23^ (1–10^5^ Hz). An EDL formation accompanied by electrochemical reaction facilitate the ion diffusion and injection into sub-layer oxide films. Following increase of capacitance in the *f* < 1 Hz is accompanied by the occurrence of a pseudo-capacitance stage (Stage ɪɪɪ), which is the indication of electrochemical doping^23^. Considering the ionic size of indium and titanium (81 pm for In^3+^ vs. 53 pm for Ti^4+^), the substantial ion intercalation into the TiO_2_ structure is thermodynamically and energetically preferable condition^25^ (Fig. 2a). However, both interstitial and substantial incorporation of In-atoms in TiO_2_ lattice were previously reported^25^. Herein, to intercalate In-ions into TiO_2_ structure, the constant *V*_*G*_ of 2 V for 60 min was imposed on the SE/TiO_2_ interface. Owing to ionic size mismatch, a structural distortion is expected upon the substantial replacement of ionic In into TiO_2_ lattice. The characteristic Raman vibration peaks of TiO_2_ were, respectively, observed in the^26^ 138, 394, 510, and 635 cm^−1^ (Supplementary Fig. 4). It was realised that in one case the B_1g_ mode of TiO_2_ at 408 cm^−1^ has shifted to 403 cm^−1^. Furthermore, the characteristic vibration peaks of In-O are detected^26^ at 307 and 366 cm^−1^, respectively, which indicates the successful In-ion intercalation into TiO_2_ sub-layers. The intercalation of In-atoms into TiO_2_ lattice structure modulated the optical properties of TiO_2_ film^27^. In-ion doping has shifted the bandgap of TiO_2_ film (3.1 eV) towards the visible light region (2.03 eV) (Supplementary Fig. 5). The proposed mechanism includes the modification of adsorption spectra of TiO_2_ via substitution of an In-atom in TiO_2_ structure (Ti_15_In_1_O_32_)^28^. The following X-ray photoelectron spectroscopy (XPS) profile measurements (Fig. 2c, Supplementary Fig. 6 and Supplementary Note 4) show 45 at. % concentration of In-atoms at top most layer of TiO_2_, which sharply declines to 4 at. % at the etching depth of 30 Å.

### Memristor devices

The potentiodynamic measurements (*I-V* sweep) of Pt/TiO_2_/Au and Pt/In-doped TiO_2_/Au memristor devices under the dark condition are presented in Fig. 2d. Pt/TiO_2_/Au device shows bipolar switching behaviour with a set and reset events at 0.94 and −0.98 V, respectively. Bipolar switching can be explained by the ionic drift of oxygen vacancies owning to employed voltage on the electrochemically inert Au and Pt electrodes^22^ (Supplementary Fig. 7 and Supplementary Note 5). This device is categorised as valence change memory (VCM) cells^22^. Noteworthy, a complementary resistive switching (CRS) behaviour is observed when the sweeping voltages are imposed on Pt/In-doped TiO_2_/Au device (Fig. 2d, Supplementary Fig. 8 and Supplementary Note 6) where the device is set at higher voltage and again reset at lower voltage of the same polarity. The identical switching behaviour was also observed at the negative bias voltage. The CRS has been demonstrated previously in metal-ion conducting bridging random access memories (RAMs) and in metal-oxide heterostructures^29^. The most plausible explanation for CRS behaviour^30^ of In-doped memristor can be the electroforming of conductive filaments of In-ions (Supplementary Fig. 8 and Supplementary Note 6). The filamentary mechanism occurs when the thermochemical active electrodes (Cu, Ag) are used. In this case the electrochemical metallisation (ECM) of the mobile cations is caused by the migration of metallic cations^22,30^. It facilitates the development of filamentary metal bridges (on state), which can be ruptured later (off state). Here, the ECM mechanism was observed in the In-doped TiO_2_ memristor device, while the inert Au and Pt electrodes are employed. From another standpoint, in heterostructured oxide stacks, the conjunction of an oxygen deficient oxide layer (low-resistance state-LRS) with another oxygen rich layer (high-resistance state-HRS) can trigger the CRS behaviour of heterostructured memristor device caused by the movement of oxygen vacancies. Based on analytical XPS measurements (Fig. 2c), the oxygen concentration in 25 Å thick upper layer of In-doped TiO_2_ film is less than the bottom layer of TiO_2_, creating a junction between deficient (LRS) and rich oxygen (HRS) components of In-doped TiO_2_ film. This gradual change of oxygen concentration can trigger occurrence of CRS of memristor due to the difference in resistance of TiO_2_ stack. Generally, CRS behaviour can be explained by both mechanisms, i.e., vacancy drift and migration of In-ions. The versatile switching behaviour of In-doped TiO_2_ device can prove that both anion and cation components are involved in CRS mechanisms. The credibility of resistive switching also demonstrates the reliability of memristor for multilevel switching operations (Supplementary Fig. 9 and Supplementary Note 7). As a key point, the loop opening is observed in *I-V* sweeping curves (Fig. 2d), which is the characteristic of charge trapping/de-trapping phenomenon. The In-doped TiO_2_-based device has bigger loop and lower bandgap (2.03 eV) than those of TiO_2_ film confirming its promising capability for application as visible light optical neuromorphic devices^31,32^.

### Synaptic devices

The two-terminal ITO/In-doped TiO_2_/AU device of Fig. 2e behaves like a biological synapse where the presynaptic signals (pulsed lights) are detected by the optical device and then postsynaptic signals are generated. By applying the similar sweeping voltage under continues illumination of *λ* = 530 nm light, the two-terminal device again shows the CRS behaviour (Fig. 2e). Here, the transparent ITO terminal does not demonstrate any electrochemical effects, which can be attributed to the low applying voltage. The increase of light intensity is accompanied by the increase of LRS current (Fig. 2e) and device conductance (Supplementary Fig. 10). Light illumination led to the decrease of *V*_Set_, which means the switching to LRS becomes easier during forward bias voltage (0 V → 1 V). It further indicates that the light illumination can affect the device conductance and assist the HRS to LRS transition. To evaluate the synaptic behaviour of optical memristor device, a pulsed light (*λ* = 530 nm LED laser) was used to stimulate artificial optical synapse. A constant voltage of 1.5 mV was employed on synaptic device. The examples of excitatory postsynaptic currents (EPSC) of TiO_2_ and In**-**doped TiO_2_ devices are demonstrated in Fig. 2f when a single 100 ms pulsed light was applied. EPSC is the photocurrent of optical synapse corresponding to the synaptic weight of its biological counterpart^33,34^. While the In**-**doped TiO_2_ synapse evidently demonstrates postsynaptic reaction to the pulsed light, the reaction of TiO_2_ synapse is ignorable (Inset in Fig. 2f). The short-term plasticity (STP) and long-term plasticity (LTP) are the conceptions in neuroscience, which can be defined based on the lifetime of postsynaptic currents^13,33^. Paired-pulse facilitation (PPF) values of devices, as the manifestation of STP, are demonstrated by two consecutive EPSC spikes with pulse intervals of 0.3 s (Fig. 2g). PPF index is defined as the ratio of amplitude of the 2nd EPSC (A_2_) to the 1st EPSC (A_1_), which is a fundamental parameter depicting temporal recognition of informative signals^13,33^. In this process, the remained photo-generated carriers of the first spike will assist the conductance of the following one. The rapid decay of PPF index with the increase of pulse intervals confirms the sensitivity of the STP to sequence of the optical pulses (Fig. 2h). The energy consumption of a singular synaptic event^35^ is calculated by *I* × *t* × *V* where *I* is the current of device, *t* is optical pulse duration and *V* is voltage, respectively. Measurements show that shorter pulse duration results in lower energy consumption (Inset of Fig. 2h). Here, the minimum consumed energy of 2.41 pJ was calculated for a synaptic event with 10 ms pulse duration without considering the energy consumption by optical laser. Supplementary Table 1 clearly shows that the considerable improvement has been achieved compared with the other current artificial oxide synapses reported-to-date, even though the energy consumption of our artificial synapse is still higher than that of human brain, which uses 1–10 fJ energy per a synaptic event^6^ (Supplementary Note 8).

The transition from STP to LTP in biological systems is the manifestation of information transfer from the short-term to long-term memory^31^. The optical synapse was stimulated by successive laser pulses with the same amplitude at the different frequencies (Fig. 2i) to imitate the STP to LTP transition in a biological system. The results demonstrate that the optical stimuli with lower pulse intervals are beneficial for facilitation the LTP capabilities. Observations confirmed that the shorter pulse intervals resulted in the higher gain values, which is consistent with the effect of residual generated carries on the following pulses (Supplementary Fig. 11a). The same behaviour is observed in the case of conductance values, where shorter pulse intervals and higher pulse numbers are equivalent to higher conductance values (Supplementary Fig. 11b). The conductance values increase rapidly at the beginning and then saturate. The capability for emulation of bidirectional analogue switching was confirmed by the observation of long-term potentiation-depression behaviour. It is facilitated by applying sequential optical pulses and then followed by imposing negative voltage pulses (Supplementary Fig. 12). The device-to-device uniformity of optical synapses was evaluated after 5 months from its initial tests. To this aim, another new sample from unscheduled part of an ALD deposited 4-inch patterned wafer was chosen and then In-Ion doping was employed. The results obtained (Supplementary Fig. 13) confirmed remarkable repeatability and device-to-device uniformity of memristor and optical synaptic devices.

### Sensorimotor device

**Fig. 3:**
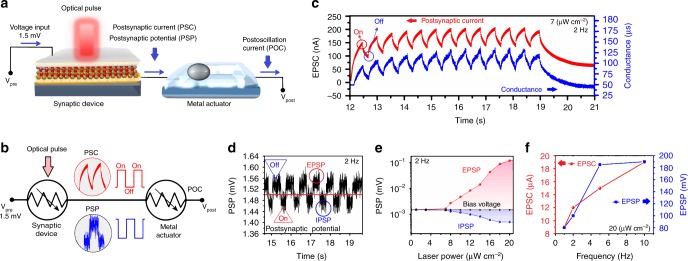
The sensorimotor system and its optoelectronic characteristics of synaptic device. **a** The scheme of sensorimotor system and **b** corresponding circuit. **c** A typical EPSC and conductance variation for ITO/In-doped TiO_2_/Au device illuminated by 7 μW cm^−2^ pulsed light (530 nm, 2 Hz) and **d** the corresponding variation of PSP of the same device. **e** The variation of PSP vs. the laser light intensity. **f** The variation of EPSC and EPSP vs. frequency of pulsed light

Overall, the sensorimotor device consists of a synapse and a metal actuator component in which the conductance of synaptic device is modulated by the optical pulses of visible light (Fig. 3a). The mechanical oscillation of the liquid metal component was facilitated by the transfer of patterned postsynaptic potential (PSP) pulses of synaptic device into liquid metal actuator. In sensorimotor, galinstan liquid droplet acts as the mechanical component. The simplified scheme of electrical circuit of sensorimotor system is presented in Fig. 3b. A bias voltage of 1.5 mV is imposed on sensorimotor device. The conductance of synapse is modulated by applying sequential *on/off* cyclic pulsed lights as the function of incident light power and frequency. The outputs of optical synaptic device including PSCs and PSPs are measured by using a source metre. The conductance of synaptic device increased after applying an optical pulse, which was simultaneously accompanied by the increase of PSC maximum (EPSC) and the decrease of PSP minimum intensities. The variations of typical PSCs, corresponding conductance and PSPs of ITO/In-doped TiO_2_/Au device are demonstrated in Fig. 3c, d, respectively. The 7 μW cm^−2^ pulsed light (2 Hz) was used as the optical source. The conductance switching of optical synapse results in the vibration of PSPs (Fig. 3d). Noteworthy, PSP oscillates continually by applying *on/off* cyclic pulsed lights, where the PSP either jumps to higher voltage (light pulse-off) or suddenly falls to lower voltage (light pulse-on) than that of the imposed bias potential (1.5 mV) (Fig. 3d). These behaviours are respectively similar to the excitatory postsynaptic potentials (EPSP) and inhibitory postsynaptic potentials (IPSP) phenomena in biological neuroscience^33,34^. It is found that the laser power has accumulative effects on EPSPs and IPSPs (Fig. 3e), i.e., the increase of power intensity is accompanied by the increase of PSP maximum and minimum values. It further shows that the nonlinear resistance of device is continuously altering with changing the light power intensity, which is the characteristic of memristor devices^36^. The polarisation of postsynaptic voltage is meaningful, since it shows that the pulsed light affected the synaptic dynamics and resistive behaviour of optical device allowing the adjustment of the synaptic responses according to the light intensity and frequency. The effect of light frequency on EPSC, EPSP and conductance of optical synaptic device are, respectively, depicted in Fig. 3f and also in Supplementary Figs. 14 and 15. The observations revealed that the increase of frequencies of the pulsed light resulted in higher EPSC values.

**Fig. 4:**
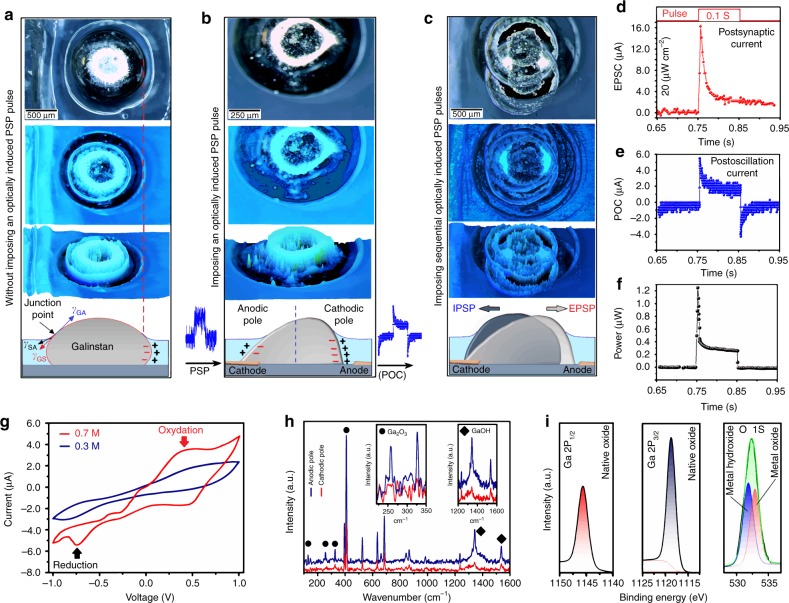
The observation and characterisation of oscillation behaviour of galinstan droplet. **a** From top to down, respectively, demonstrates the actual top view, the optical contrast image, the generated 3D model and schematic interpretation of a galinstan droplet inside NaOH bath, without imposing applied potential, **b** when a singular postsynaptic pulse of 50 ms laser pulse (20 μW cm^−2^) was transmitted to galinstan actuator, and **c** when the consecutive laser pulses are used to oscillate the galinstan actuator. **d** Shows the EPSC of synaptic device stimulated by 0.1 s pulsed laser (20 μW cm^−2^), and **e** the corresponding POC of galinstan actuator, and **f** corresponding power consumption for oscillation of galinstan actuator. **g** The *C-V* of HGDE device. **h** The results of in-situ Raman studies of galinstan droplet. Inset shows the peaks of Ga_2_O_3_ and GaOH. **i** The core level XPS spectra of Ga2P, and O1S of surface oxide of galinstan droplet

Here, the sinusoidal PSPs have been innovatively employed as the driving force to oscillate galinstan droplet in NaOH bath. The electro-capillarity, which is alteration of the surface energy of a liquid droplet by using an applied potential^37^, was used to change the physical configuration of liquid metal. To clarify the principle of mechanical oscillation, a galinstan droplet was semi-submersed into NaOH solution (0.7 M) and its oscillation was monitored by using an optical microscope with the capability of fast imaging and reproduction of three-dimensional (3D) models. The system works based on the optical contrast of surfaces and then incorporates several images in one single file. Figure 4a (from top to down) respectively demonstrates the actual top view, the light contrast developed image, the 3D model and finally the scheme of galinstan droplet. When a galinstan droplet is semi-submersed into NaOH solution (electrolyte), the surface of electrolyte shifts downward in air/solution/galinstan junction point and decorates a concave interface^38^ (scheme in Fig. 4a). It happens since the surface energy of galinstan/solution (*δ*_GS_) is higher than that of galinstan/air (*δ*_GA_) and that of solution/air (*δ*_SA_)^38^. The tie-line in Fig. 4a shows the boundary between the galinstan droplet and the NaOH solution. From the charge distribution point of view, the surface of immersed part of galinstan droplet in NaOH solution is negatively charged. Consequently, the positively charged ions are accumulated at the diffuse electric double layer (EDL) at galinstan/NaOH solution interface^39^. At the absence of the external potential, the initial charges (*q*_*0*_) are distributed uniformly throughout the interface. The amplitude of *q*_*0*_ is related to the electrolyte concentration and the size of galinstan droplet. By applying a singular external potential, the galinstan/NaOH system acts as an electrolyte cell. When a PSP pulse is applied to galinstan droplet (scheme in Fig. 4b), the charge distribution on the droplet surface will stay uniform, which is facilitated by the high conductivity of galinstan^39^. However, since the conductivity of electrolyte is limited, a potential gradient will be expanded throughout the electrolyte, which is followed by generation of the charge gradient on the galinstan/electrolyte interface. In this condition, the voltage drop across the EDL on anodic pole of droplet results in higher surface tension and pressure on the anodic half of droplet according to the Lippmann equation^39^ (Supplementary Note 9). Owing to the pressure difference between two halves of galinstan droplet and its surrounding electrolyte, an imbalance is caused inside of galinstan droplet, which leads to its mechanical deformation^39^. These phenomena are also similar to depolarisation-repolarisation and hyperpolarisation characteristics of biological cells^33,34^, which are fundamental functions for communication between the biological cells. In Fig. 4b, an accurately captured image depicts the exact moment of deformation of conical head of galinstan droplet, which is inclined to the cathodic pole. The mechanical deformation continues until the pressure difference drops on both sides of galinstan and system returns to its equilibrium condition. This mechanical deformation is called continuous electrowetting (CEW)/de-electrowetting (DEW), which is famous for its low-power consumption^40^. CEW is originated from the existence of surface tension gradient between the galinstan droplet and its surrounding NaOH solution. Figure 4c shows the vibration of a galinstan droplet after receiving sequential PSP pulses. Here, for the first time, optically generated PSPs were employed to emulate the heartbeat oscillation of galinstan droplet. To oscillate a 1200 μm diameter galinstan droplet with the estimated height of *h* ~500 μm, a pulsed light with power density of 20 μW cm^−2^ was employed. It was found that the amount of input current into metal oscillator (i.e., EPSC) is different to the amount of output current after oscillation of liquid droplet (i.e., postoscillation current or POC). The power consumption for a singular heartbeat oscillation caused by 0.1 s optical pulse was calculated. The difference between EPSC (Fig. 4d) and POC (Fig. 4e) and voltage variation (*V*_Max_–*V*_Bias_) were used to calculate the required power consumption for oscillation of galinstan droplet (*ΔI* × *ΔV*). Regarding the voltage variation, it was found that a singular oscillation, activated by 0.1 s pulse duration, by average needs 1.2–0.3 μW power (Fig. 4f) and approximately consumes 30 nJ energy.

The oxidation and re-oxidation (Redox) of native surface oxide of galinstan in electrolyte solution is highly dependent on the electrolyte concentration. This redox reaction has facilitated the mechanical oscillation and even the flow of liquid metal. The growth of gallium oxide on the surface of galinstan is described by^41^:

1 $$2{\mathrm{Ga}} + 3{\mathrm{H}}_2{\mathrm{O}} \to {\mathrm{Ga}}_2{\mathrm{O}}_3 + 6{\mathrm{H}}^ + + 6{\mathrm{e}}^ -$$

However, NaOH solution dissolves gallium oxide to produce 2NaGa(OH)_4_ (Formula 2), which assists the formation of hydrophilic interfaces^41^. It results in lower interfacial tension between the electrolyte and liquid metal, and consequently, the galinstan droplet would be capable of spreading and movement:

2 $${\mathrm{Ga}}_2{\mathrm{O}}_3 + 2{\mathrm{NaOH}} + 3{\mathrm{H}}_2{\mathrm{O}} \to 2{\mathrm{NaGa}}\left( {{\mathrm{OH}}} \right)_4$$

A hanging galinstan drop electrode (HGDE) with different NaOH concentrations was designed (Supplementary Fig. 16) to investigate the effect of NaOH concentration on the surface oxide of galinstan. In the cyclic voltammetry curves (Fig. 4g), redox reactions appear only when the concentration of NaOH solution reaches 0.7 M. Thus, this specific electrolyte concentration was used in sensorimotor device. An in-situ Raman measurement technique, as a viable method for the characterisation of surface components of liquid alloys^42^, was organised to characterise the surface materials of the galinstan droplet on each individual anodic and cathodic poles during heartbeat oscillation (Fig. 4h and Supplementary Fig. 17). The study of Raman spectra interestingly shows that the anodic pole has strong Raman characteristic vibration of Ga_2_O_3_ (136, 256, 326 and 413 cm^−1^) and GaOH (1342, 1536 cm^−1^), while those peaks are weak or absent on the cathodic pole. These behaviours can be explained by the variation of polarised PSPs, which act like a sinusoidal potential source. The XPS measurements on mechanically exfoliated surface film of the galinstan droplet (Fig. 4i and Supplementary Fig. 18) depict the Ga 2P_1/2_ and Ga 2P_3/2_ peaks of native Ga_2_O_3_. The detection of deconvoluted O 1S peak in both native gallium oxide and gallium hydroxide confirms that the Ga_2_O_3_ and GaOH are the main components of surface film of galinstan droplet.

### Mechanical oscillation of metal actuator

**Fig. 5:**
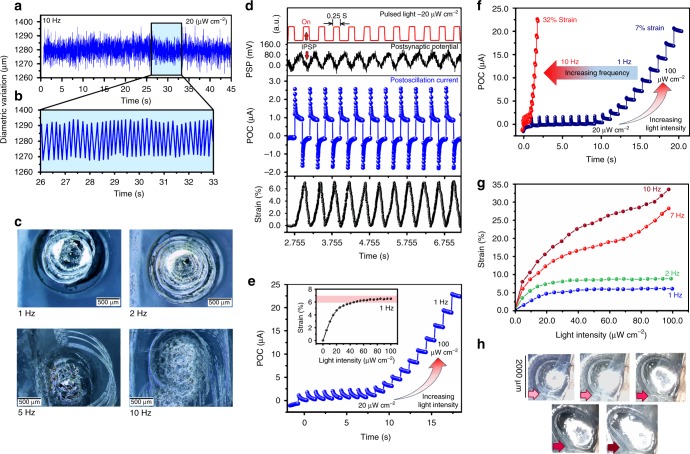
The modulation of oscillation of the galinstan droplet. The **a** and **b** are the typical transitional motion of a 1280 μm diameter galinstan droplet by applying sequential PSP pulses originated from the optical synapse. **c** The typical optical images show the oscillation of galinstan droplets with various frequencies. **d** From top to down, the graph of patterned pulsed light, variation of PSP and POC and its corresponding mechanical strain. A 20 μW cm^−2^ pulsed laser (530 nm) with 2 Hz frequency and 250 ms intervals are employed. **e** The variation of POC and corresponding strain of liquid metal actuator when the light power is increasing from 20 to 100 μW cm^−2^ at constant frequency of 1 Hz. **f** The variation of POC of liquid metal droplet, when the combination of increased light intensity (from 20 to 100 μW cm^−2^) and increased pulse frequency caused unrested strain in galinstan droplet. **g** The effect of light intensity and frequency on mechanical strain of galinstan droplet. **h** Optical images demonstrate the sequential size expansion of galinstan droplet (strain is sequentially increasing from left to right)

It is aimed to achieve the controlled oscillation of galinstan droplet by using optically generated sequential pulses, in which the dimension of oscillator droplet either returns to its initial size after each individual expansion or enlarges continually. Series of systematic experiments were carried out to evaluate the capability of our device as an artificial sensorimotor system and to investigate the relation between sensory functions and mechanical strain of droplets. In doing so, the PSPs were generated using pulsed light with the different intensities and frequencies and then were transmitted to the galinstan droplet. The outstanding electrochemical feature of galinstan droplets allows the oscillation of them by using sequential voltage pulses. As an example, typical 20 μW cm^−2^ visible light pulses with 10 Hz frequency were used to stimulate the optical synapse to generate PSP pulses. The displacement of the border lines of ~1280 μm diameter galinstan droplet were recorded during oscillation (Fig. 5a). The oscillation monitoring was fulfilled by analysis of the optical microscopic images (Supplementary Note 10 and Supplementary Fig. 19). The sinusoidal PSPs caused the stable periodic expansions and contractions of galinstan droplet (Fig. 5b).

It is also possible to employ optically generated PSPs to tune the oscillation of the galinstan droplet over a range of vibration frequencies from 1 to 10 Hz and finally have no remained strain after each individual oscillation (Fig. 5c). Figure 5d from top to down demonstrates how the stimulation of optical synaptic device (ITO/In-doped TiO_2_/Au) by the sequential pulsed light (20 μW cm^−2^) with 250 ms pulse duration and 250 ms pulse intervals has produced PSP signals. Noteworthy, the IPSP phenomenon is also observed at all frequencies. The POC variations (output current after oscillation of galinstan) were measured and shown in Fig. 5d. It is observed that the POC followed a declining pattern during employment of an individual PSP pulse and even dropped to the negative values. This is attributed to the polarisation of PSPs during *on/off* cyclic pulsed light and the IPSP phenomenon. The strain measurement revealed that the size of galinstan droplet returned to the initial size after each oscillation (Fig. 5d). In our investigation, the galinstan droplet has experienced ~7% strain after receiving PSPs signals generated by optical pulses with 250 ms pulse duration and 250 ms pulse intervals. Here, it was found that the 1 cm^2^ optical synaptic device could generate enough potential energy to vividly oscillate the galinstan droplet with 1500 μm diameter when it was illuminated by pulsed light with the minimum intensity of 20 μW cm^−2^. Supplementary Movies 1 and 2, respectively, demonstrate the optically induced heartbeat oscillation of a typical galinstan droplet, which is stimulated by a series of optical pulses with 1 s and 3 s durations.

Nevertheless, it was observed that the increase of light intensity alone cannot facilitate continues size expansion during heartbeat oscillation of the galinstan droplet. For example, the 1 Hz pulsed light can cause 6–7% temporal strain, even the light intensity is increasing continually from 20 to 100 μW cm^−2^ (Fig. 5e). It was discovered that at shorter pulse intervals and higher light frequencies, the POCs do not return to the initial level (Fig. 5f). In this case, the galinstan droplet acts as another synaptic component in the system where the charge transfer through the galinstan/liquid electrolyte interface controls the output current of sensorimotor system (POC). Such synaptic behaviour could either be accompanied by unrested strain after each individual oscillation of galinstan droplet or be merely restricted to temporal oscillation. It was found that the combination of increasing light power and frequency assisted continues mechanical deformation of the galinstan droplet (Fig. 5g). To have the state of continues size expansion, higher frequencies of pulsed light at stronger light intensities are necessary to be employed. The optical microscopic images in Fig. 5h depicts continues expansion of galinstan droplet (from left to right), where the 35% strain was finally measured after imposing 100 optical pulses (100 μW cm^−2^) with 10 Hz frequencies. These observations collectively depict the capability of the sensorimotor system as a practical platform for development of optically stimulated artificial mechanical systems, which mimic the contraction/expansion performance of a sensorimotor muscular system after receiving postsynaptic action potential pulses.

## Discussion

The present study demonstrated the capabilities of the artificially developed bioinspired optoelectronically sensorimotor device with optical synaptic and actuating components to mimics the functionalities of opto-genetically engineered biological motor systems. In fact, the developed optically stimulated synapse is a memristor device with a visible light-sensitive component, which has facilitated various synaptic dynamics. The outputs of the optical synapse were intentionally patterned and employed to oscillate a liquid galinstan droplet, which can emulate both the expansion and contraction of the fibre muscles at different frequencies. The characterisation studies revealed that the potentially activated electrochemical mechanisms are behind the oscillation of liquid galinstan droplet. The combination of several optoelectronic functionalities, synaptic properties, fluid dynamics and materials science enabled us to decorate an internet of things of bioinspired optoelectronic sensorimotor device with the low-energy consumption and stable reliable performance. This device has profound impact on numerous applications in artificial neurological sensorimotor, light-driven neurorobotics, microfluidic chips and micromechanical pumps in drug delivery systems.

## Methods

### Fabrication and characterisation of optical synapses

Three individual Au electrodes with 100 μm gap (source-drain) and 200 μm gap (gate-source) were patterned by electron beam (EB) evaporation on the Si/SiO_2_ substrate (Supplementary Fig. 2). Plasma enhanced atomic layer deposition (PE-ALD) was employed to deposit 7 nm thick TiO_2_ films over Au source-drain electrodes. The Ti(N(CH_3_)_2_)_4_ (Strem Chemical) precursor and O_2_ plasma were used in PE-ALD process. To intercalate In-ions into TiO_2_ film a solid electrolyte (0.1 M InCl_4_ aqueous solution dissolved in polyethylene glycol diacrylate) was deposited over TiO_2_ films and Au electrodes by drop casting (Supplementary Fig. 2). To intercalate In-ions into TiO_2_ film a constant *V*_*G*_ of 2 V for 60 min was imposed on SE/TiO_2_ interface. To drive *V*_*G*_ and measure source-drain current a two-terminal Keighley 2614B source metre was used. The scan speed of source metre was 10 mV s^−1^. The capacitance measurements were performed at the frequency ranges from 10^6^ to 10^−1^ Hz by using an Autolab Metrohm (PGSTAT204). After In-ion intercalation, the In-doped TiO_2_ films over Au electrode were used for the following experiments. To fabricate memristor devices, 60 nm Pt film was deposited by sputtering over Au-TiO_2_ film to finally fabricate Pt-TiO_2_-Au heterostructures (Supplementary Fig. 2). With the same method, 60 nm indium tin oxide (ITO) transparent film was also deposited on the Au-TiO_2_ film to fabricate the optical synapses (Supplementary Fig. 2). To measure the synaptic behaviour, a tunable LED laser (*λ* = 530 nm) driver in combination with Autolab (PGSTAT204) was employed to precisely design and pattern consecutive pulses.

### Fabrication of liquid metal actuator

To fabricate the mechanical actuator component of sensorimotor system, a 125 mm^3^ polydimethylsiloxane (PDMS) chamber with source and drain tungsten electrodes was developed over glass substrate. Six milligram liquid metal galinstan droplet (68.5% gallium, 21.5% indium, 10% tin) was located on the source electrode in chamber. The exact volume of galinstan was measured and transferred by pipette and then was located on prescheduled position under optical microscope to precisely seat galinstan droplet on source electrode. NaOH aqueous solution (0.7 M) was prepared for electrochemical cell. The galinstan droplet was semi-submersed by NaOH electrolyte in the PDMS chamber.

### Materials characterisation

Various characterisation techniques were employed for investigation of the materials properties and for measurement of the mechanical replacement of galinstan metal actuator during oscillation. Optical microscope (Olympus SZX 16) facilitated by the digital camera with the capability to record 20 frames per second during in-situ observations was employed to take the images of galinstan droplet during oscillation. A 3D model was developed by software (iSolution Lite X64*)* based on the optical contrast of surfaces. The prepared image was processed to measure the area of galinstan droplet during oscillation to estimate their mechanical strain (Supplementary Fig. 19).

To characterise the materials on the surface of galinstan droplet during oscillation, a custom made system was designed and fabricated in which the metal actuator system was monitored during the oscillation. During in-situ Raman measurements (Supplementary Fig. 17) the anodic and cathodic poles of galinstan droplet were individually targeted by continues laser beam of Raman (*λ* = 750 nm, HORIBA micro-Raman, Lab Ram ARAMIS). In the case of TiO_2_ film, Raman measurements were performed by the regular method. XPS (Thermo Scientific theta probe) was utilised to analyse the composition of In-doped TiO_2_ film. To measure the atomic percentage of individual elements in TiO_2_ film and to estimate the diffusion depth of In-atoms, the XPS depth profile was provided. The mechanical exfoliation was used to separate the surface oxide of galinstan droplet from their host galinstan alloy to investigate their chemical compositions. To this aim, the electrolyte bath was depleted from the liquid and then a quartz sample was used to separate the surface oxide film by mechanical exfoliation (Supplementary Fig. 18). The absorbance and reflectance spectra of samples were measured using UV-visible diffused reflectance spectrometer (Shimadzu, UV-2600).

### Hanging galinstan drop electrode

A hanging galinstan drop electrode (HGDE) was designed to investigate the electrochemical interaction between the alkali electrolyte and the surface oxide of galinstan (Supplementary Fig. 16). To this aim, a HGDE electrode, as working electrode was semi-submersed into NaOH solutions by using a Pt syringe. An Ag/AgCl miniature electrode acts as a reference electrode, and Pt wire was employed as a counter electrode, respectively. The electrochemical measurements were carried out by Autolab Metrohm (PGSTAT204).

## Supplementary information

Supplementary Information

## Data Availability

The data supporting the findings of this study are available within the article and the associated Supplementary materials. Any other data are available from the corresponding author upon request.
