# Supplementary material for: A bioinspired optoelectronically engineered artificial neurorobotics device with sensorimotor functionalities
Source: Nat Commun. 2019 Aug 27;10:3873. doi: 10.1038/s41467-019-11823-4 (PMC6712026; doi:10.1038/s41467-019-11823-4)
Supplement: Supplementary file 1 — Supplementary Information [file 41467_2019_11823_MOESM1_ESM.pdf]

Supplementary Files

A Bioinspired optoelectronically engineered artificial neurorobotics device with sensorimotor functionalities

Karbalaei Akbari[1,2] and Zhuiykov[1,2]

[1] Center for Environmental & Energy Research, Ghent University Global Campus, 21985, Incheon, South Korea.

[2] Department of Green Chemistry and Technology, Faculty of Bioscience Engineering, Ghent University, 9000 Ghent, Belgium.

* - Corresponding authors: mohammad.akbari@ugent.be; serge.zhuiykov@ugent.be

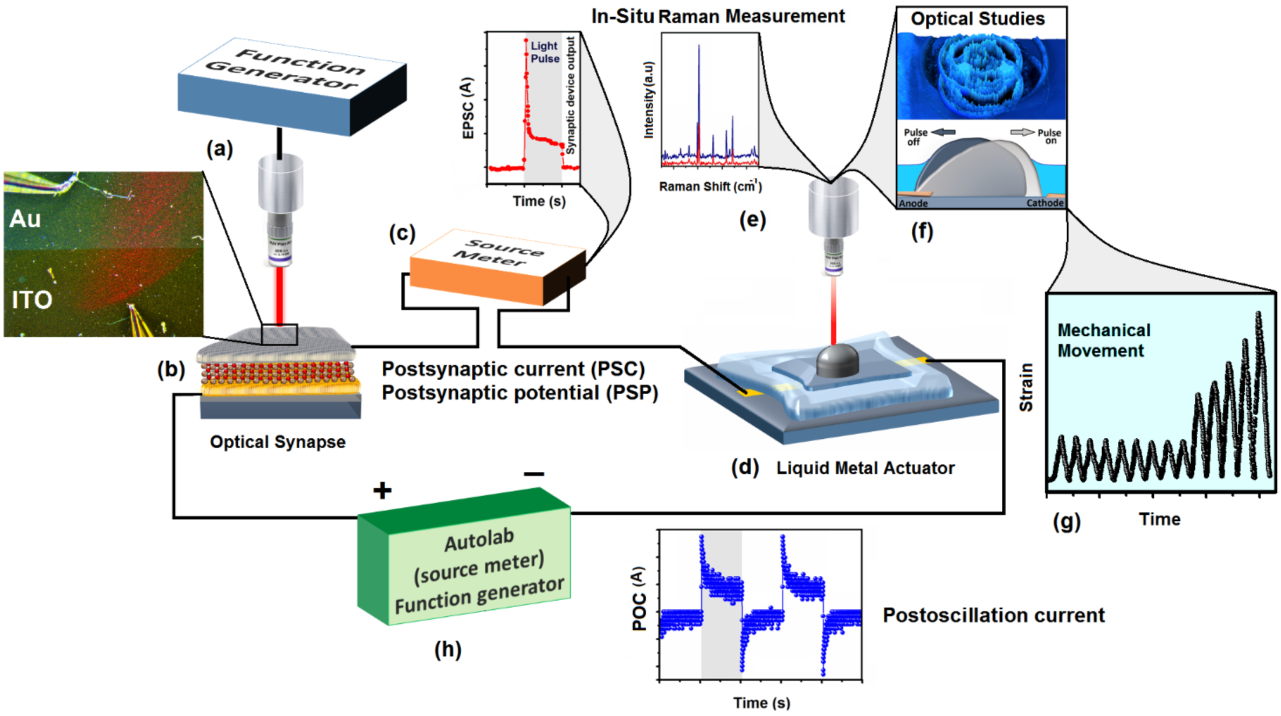

Supplementary Figure 1. The schematic representation of sensorimotor system accompanied by characterization tests and electrical measurements.

Supplementary Note 1. In setup, (a) the light pulses are patterned by a function generator to (b) illuminate the In-doped TiO_2 film and consequently change the conductance of ITO/In-doped TiO_2/Au optical synaptic device. (C) The outputs of the synaptic device including the postsynaptic currents (PSC) and postsynaptic potentials (PSP) were measured by source meter. The generated postsynaptic electrical pulses are transferred to the (d) liquid metal actuator component of sensorimotor system which consists of a galinstan droplet located in the bath of 0.7 M NaOH solution. (e) The In-situ Raman studies were employed to monitor the oxidation and reduction of the surface oxide of liquid metal galinstan during consecutive heart-beat oscillations of liquid metal actuator. (f) An optical microscope is utilized to record the oscillation of liquid metal galinstan droplet. (g) The mechanical strain of liquid metal is measured based on the calculation of the surface area of liquid metal droplets before, after and during the oscillation. (h) The electrical characteristics of sensorimotor device were measured by Autolab source meter.

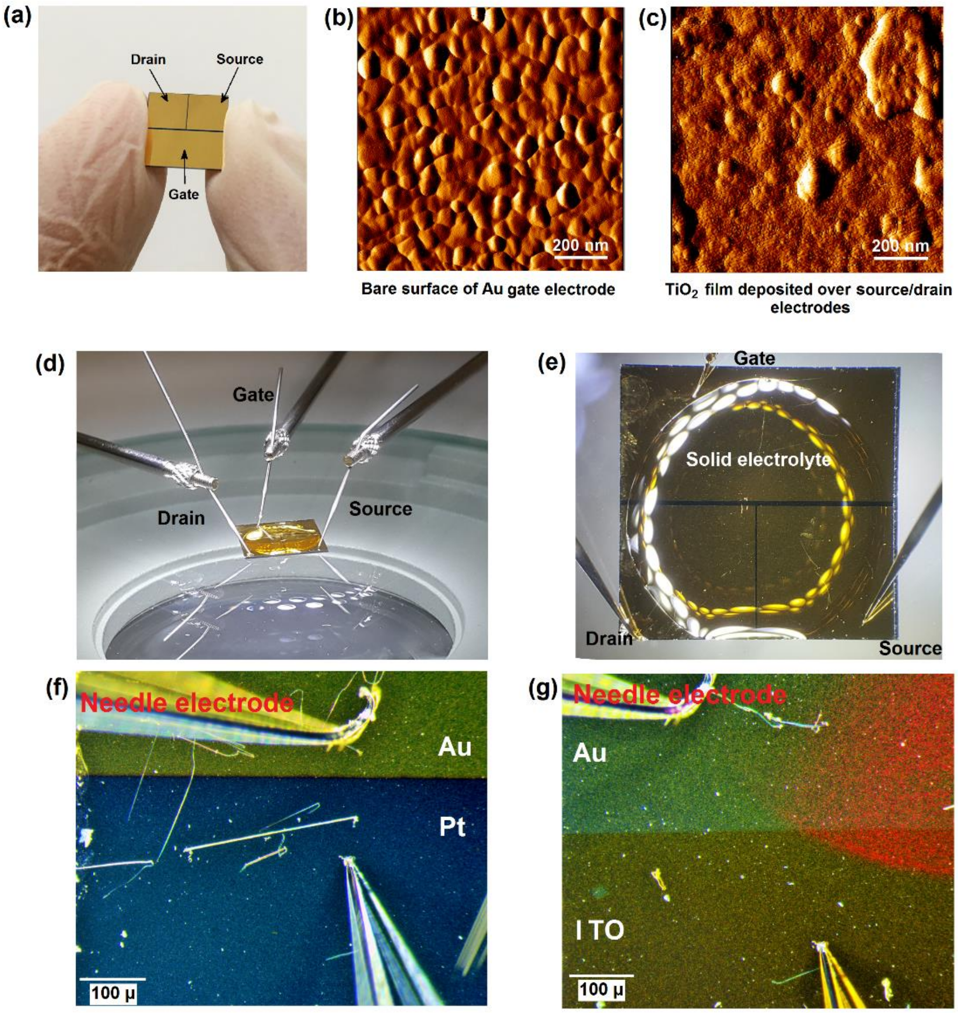

Supplementary Figure 2. (a) The three electrodes setup. (b) The AFM image shows the bare surface of Au gate electrode. (c) The AFM image shows ALD TiO_2 film on source and drain electrodes. (d) The three electrodes setup for In-ion doping in TiO_2 film and (e) corresponding enlarged image which shows gate, source, drain and solid electrolyte. (f) Top view optical image of Pt/TiO_2/Au and (g) the illuminated surface of ITO/TiO_2/Au devices.

Supplementary Note 2. By using a hindrance mask during ALD process, specific locations on the surface of Au electrodes (Gate, source and drain) were kept uncoated. Then, the In-ion incorporation process was performed on the 7 nm thick TiO_2 film deposited over the source-drain electrodes. To this aim, bias voltage was imposed on gate electrode and subsequently on SE/TiO_2 interface (Figure 2d and 2e). After ion intercalation, the SE was removed from surface and the second top electrodes (platinum in the case of memristor shown here in Figure 2f and ITO in the case of optical synapse as demonstrated in Figure 2g) were deposited on the In-doped TiO_2 film which also have Au back electrodes (substrate). Thus, finally sandwiched structures with an Au back electrode, TiO_2 semiconductor film (doped or non-doped), and Pt or ITO top electrodes were fabricated.

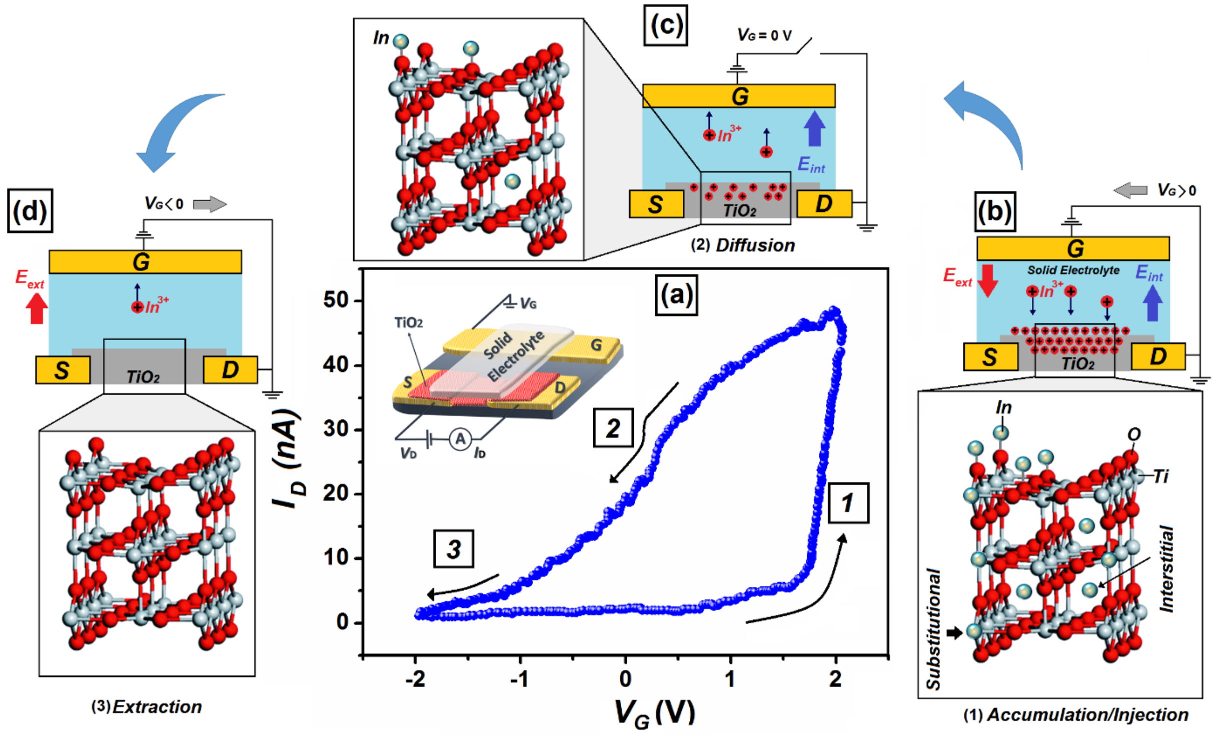

Supplementary Figure 3. The graphical scheme and electrical measurements during In-ion intercalation.

Supplementary Note 3. Figure 3 shows the variation of channel current (I_D) vs. the gate voltage (V_G). The scheme shows the device structure. (b) In-ion are pushed to accumulate at the surface of TiO_2 film surface under the positive gate voltage, which is accompanied by the development of the concentration gradient field (E_{int}). It finally results in an increase in channel conductance of TiO_2 film. By further increase of V_G, In-ions would overcome the surface diffusion and the bulk diffusion barriers to inject In-ions into TiO_2 sublayer. (c) The sweep of voltage from the upper limit (2 V) to lower voltages creates an internal field (E_{int}) which is accompanied by Ion concentration gradient[1]. The non-equilibrium distribution of ions causes concentration gradient in TiO_2/solid electrolyte interface. It roles as driving force to extract ions from oxide layer toward the SE/TiO_2 interface. When the V_G=0, the chemical potential gradient will cause an E_{int}, consequently the In-ions are driven back into solid electrolyte. (d) A negative V_G will extract the accumulated In-ions from TiO_2 film. The ion extraction progress is depended on the stability of intercalated ions in the host oxide layer[1].

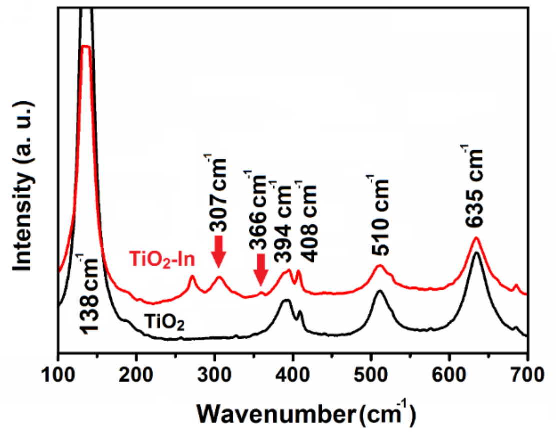

Supplementary Figure 4. The Raman characteristic vibration of TiO_2 and the In-doped TiO_2.

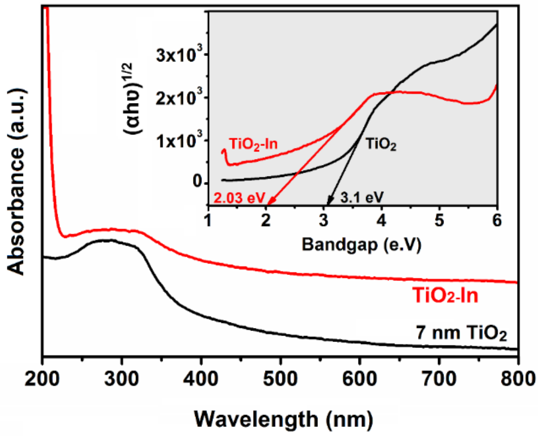

Supplementary Figure 5. The absorption spectra and bandgap values of TiO_2 and the In-doped TiO_2 films.

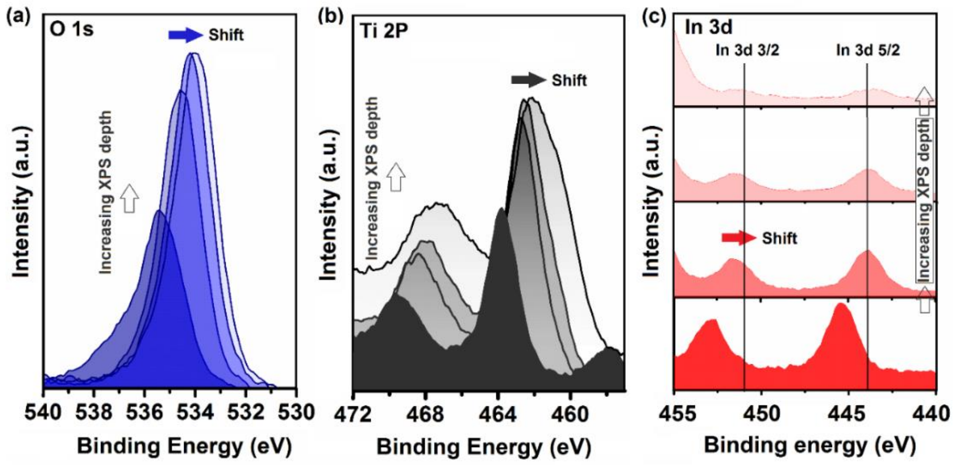

Supplementary Figure 6. The (a) O1S, (b) Ti 2P and (c) In 3d XPS spectra of In-doped TiO_2.

Supplementary Note 4. The sequential XPS depth profile measurements determine the chemical composition of TiO_2 film. Since the electronegativity of indium is higher than titanium, the intercalation of In-ions into TiO_2 film is accompanied by a positive shift to higher binding energies.

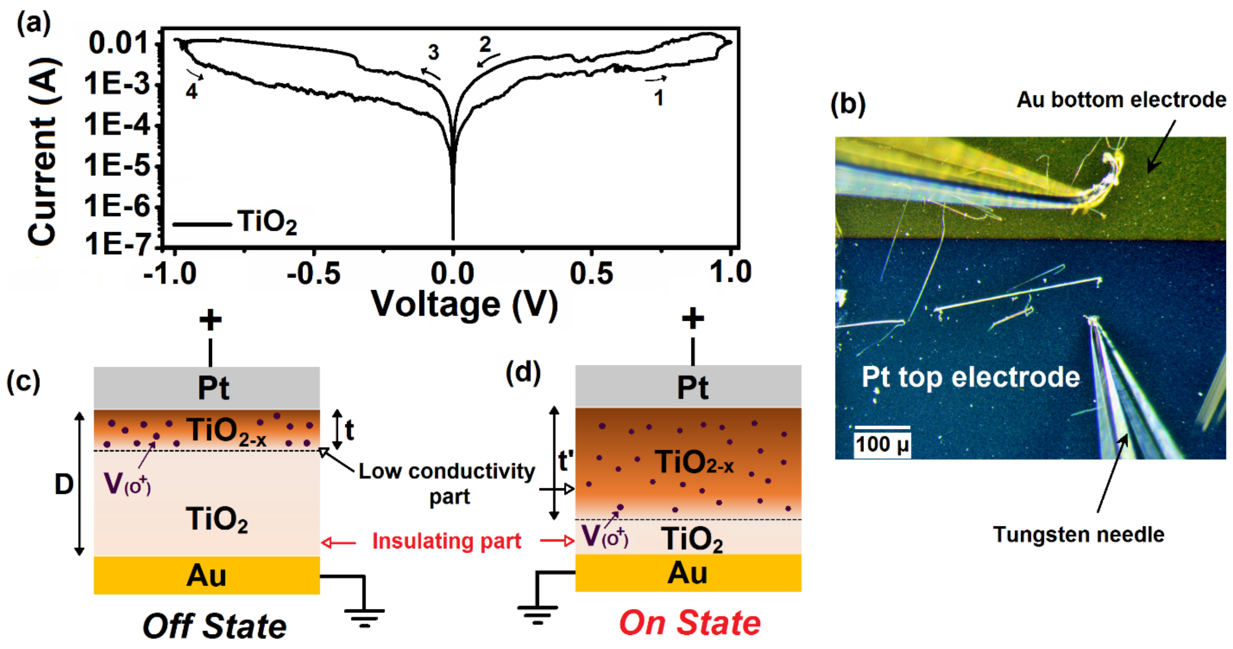

Supplementary Figure 7. Typical logarithmic scale of the *(I-V)* sweeping curves for Pt/TiO_2/Au.

Supplementary Note 5. The set and reset process were measured on atomic layer deposited (ALD) TiO_2 layer sandwiched between Au and Pt films. Before switching, all samples were formed into their low resistive switching (LRS) by applying a -3 V at a current compliance of 1 mA. (a) The typical bipolar switching curves obtained during sweeping from 0→1→0→-1→0 in the case of Pt/TiO_2/Au samples. The V_{set}=0.94 and V_{Reset}=-0.98 were measured during *I-V* sweeping test. The expected performance was observed during *I-V* sweeping in which the cell was set to a LRS at the positive voltage and reset to HRS at the negative voltage, well-known as counter-eight-wise-switching mechanism[2]. (b) The top view of Pt/TiO_2/Au memristor device. (c) and (d) demonstrate the schematic representation of oxygen vacancies distribution for both *off* and *On* states. The dielectric member of memristor with thickness of *D* has two parts. The low conductive TiO_{2-x} with the thickness of *t* and insulating part with the thickness of *D-t*. The applied voltage on the electrode, cause ionic drift of oxygen vacancies which is accompanied by the border shift between low conductivity and insulating parts. In this condition the *I-V* curve of device is not further Ohmic and will be non-linear. In this proposed model the thickness of insulating layer should be small enough (few nanometer) to let the electric field develops and promotes the ionic drift of defects.

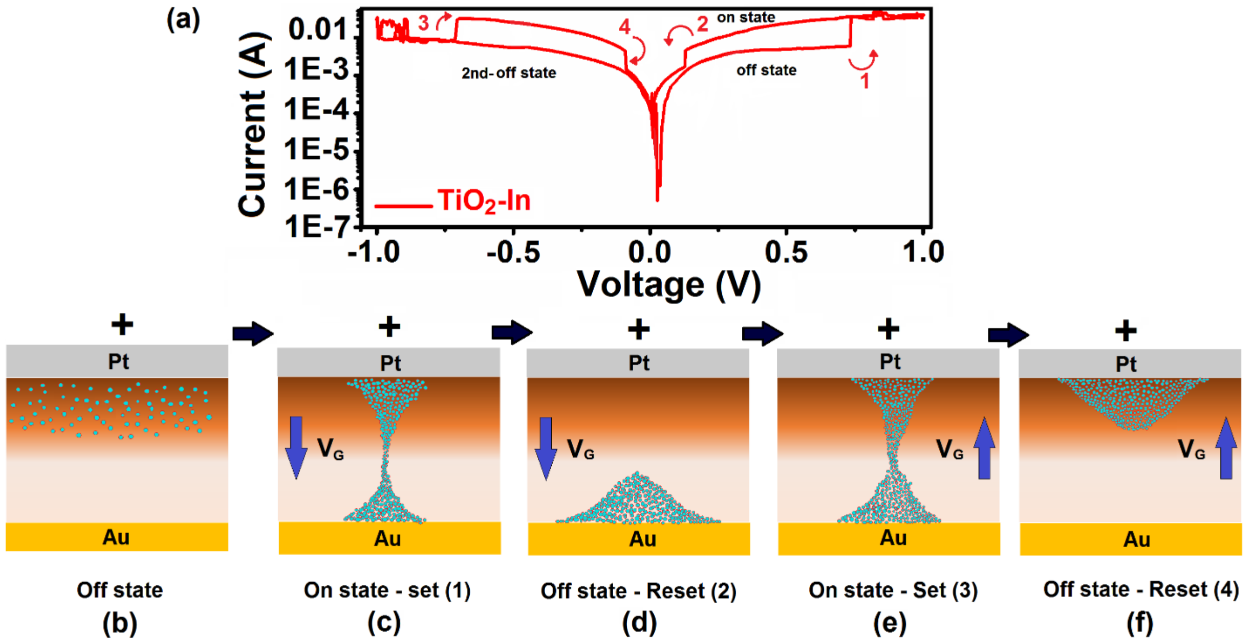

Supplementary Figure 8. (a) Typical logarithmic scale depiction of *I-V* sweeping graph of Pt/In-doped TiO_2/Au memristor.

Supplementary Note 6. In the case of Pt/In-doped TiO_2/Au device, the switching mechanism is different. A complementary resistive switching (CRS) phenomenon was observed during voltage sweeping (Figure 8a), where the cell set for the first time at V_{Set1}=0.732 V and then reset again in the same polarity at V_{Reset1}=0.1277 V. In the negative voltage, the 2nd LRS to HRS switching occurred at V_{Set2}=-0.7055 V and again reset to HRS at V_{Reset2}=-0.0901 V. Two set and reset events occurred almost at the same absolute voltage, indicating a thermally assisted mechanisms. Since two set and reset events occurred almost in the same absolute voltage, thermally assisted mechanism is one of the most possible theory which can explain the memristor behavior. The unipolar switching confirms the filamentary nature of resistive switching. The process can be explained according to the formation of conductive filaments during electroforming process which is necessary for following switching phenomena. The CRS behavior can be explained by the movements of both charged species including oxygen vacancies and cations. The redistribution of oxygen vacancies and Indium ions inside TiO_2 layer can facilitate resistive switching. At the initial state or *off* state (Figure 8b), the top In-doped layer (which also is an oxygen deficient layer) is in LRS mode while the bottom layer in HRS. By applying the threshold voltage, the ionic species migrate into lower insulating layer resulting in the formation of conducting filaments and paths (Figure 8c) and then device reaches its *on* state (*Set1*). At higher positive voltage, the ionic species in upper layer (mostly In-ions) will be depleted (Figure 8d) and again device will reach its *off* state (*Reset 2*) and demonstrates HRS behavior. The similar phenomenon is expected to occur at the negative biases in which the first *on* state (Figure 8e) switches the memristor to new LRS, following another *off* state after depletion of the ionic species (Figure 8f). The versatility of CRS behaviour of all samples was repeatedly tested several times and the same behaviour was observed.

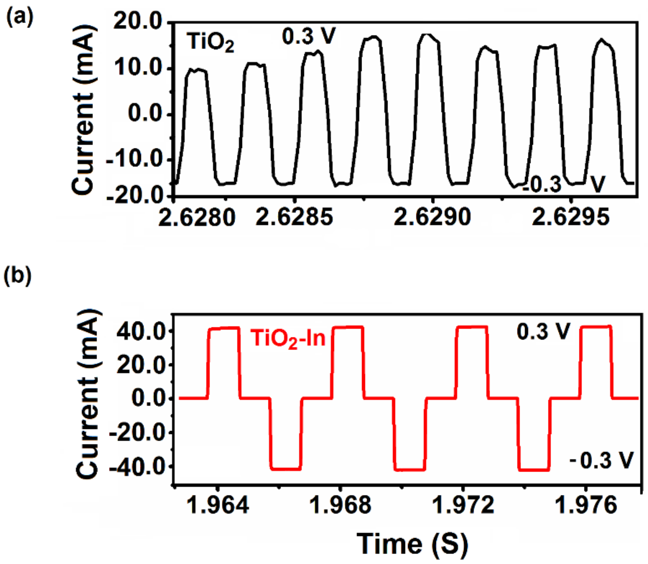

Supplementary Figure 9. The pulse driven element operation of (a) Pt/TiO_2/Au and (b) Pt/In-doped TiO_2/Au devices.

Supplementary Note 7. In order to evaluate the CRS behavior of memristor, pulse driven tests were performed. The consecutive read and write voltages was employed on the devices. The amplitude of read voltage should be selected a number between the values of V_{Set} and V_{Reset}, otherwise the information storage would not be possible. In so doing, consecutive pulses of 0.3 V and -0.3 V were imposed on the Pt/TiO_2/Au memristor device. A large current was driven, which confirms that *on/off* switching phenomenon occurred. The patterned voltages were imposed on the Pt/In-doped TiO_2/Au devices (0→0.3→0→-0.3→0 volts). It was found that Pt/In-doped TiO_2/Au device shows more uniform and sharper current peaks compared with those of Pt/TiO_2/Au memristor. The possible explanation can be attributed to facile ionic movement of In-ions in In-doped TiO_2 memristor.

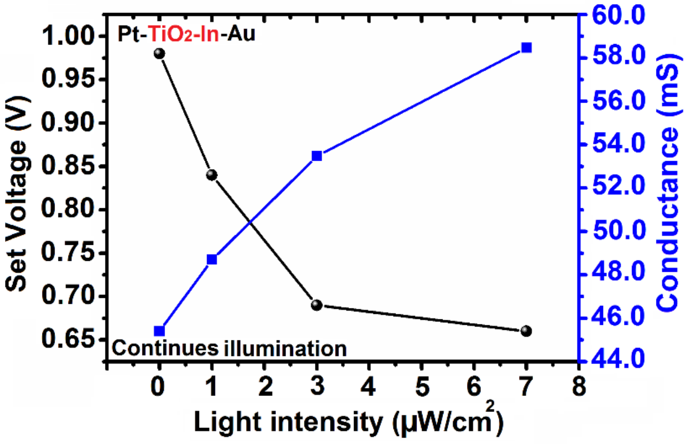

Supplementary Figure 10. The variations of V_{set} and conductance vs. the light intensity under continues illumination of λ=530 nm visible light with power density of 7 µW cm^-2.

Materials	Explanations	Driving source	Operation energy	Ref.
Human Synapse	Biological	Ionic pulses	10 fJ	3
IZO	Electrochemical (Transistor)	Voltage Pulse	45 pJ	4
ZnO_x	Ta_2O_5 as electrolyte (Transistor)	Voltage Pulse	35 pJ	5
α-MoO_3	Ionic liquid electrolyte (Transistor)	Voltage Pulse	9.6 pJ	6
α-MoO_3	$LiClO_4$/PEO electrolyte (Transistor)	Voltage Pulse	0.16~1.8 pJ	1
CuO/Ta_2O_5	Oxide heterostructures	Voltage Pulse	2.3 pJ	7
CMOS	Analogue, 180 nm transistor	Voltage Pulse	100 pJ	8
CMOS	Digital, 28 nm transistor	Voltage Pulse	25 pJ	8
CMOS	Digital, Programmable	Voltage Pulse	10 nJ	8
TiO_x/HfO_x	Resistive change	Voltage Pulse	0.85-24 pJ	9,10
TiO_x	Resistive change	Voltage Pulse	200 nJ	11
WO_x	Resistive change	Voltage Pulse	40 pJ	12
PCMO	CMOS-RRAM	Voltage pulse	6-600 pJ	13
$HfO_x/TiO_x/HfO_x/TiO_x$	Analogue	Voltage Pulse	40 µJ	14
$HfO_x/TiO_x/HfO_x/TiO_x$	Various initial resistance, CMOS - 16,348 RRAM device	Voltage Pulse	0.85 pJ -24 pJ	15
In_2O_3/ZnO_2	Oxide heterostructures	Optical Pulse	0.2 nJ	16
TaO_x/TiO_2	CMOS - 3D RRAM device	Voltage pulse	10 fJ	17
In-Doped TiO_2	Ion-doped memristor	Optical Pulse	2.41 pJ	Present study

Supplementary Table 1. The operation energy of selected metal-oxide based synaptic devices.

Supplementary Note 8. The operation energy of selected metal oxide-based synaptic devices are presented here. In most cases, an external applied voltage was employed to stimulate the synaptic device. The energy consumption of In-doped TiO_2 optical device of present study is one of the most efficient metal-oxide based synaptic devices. Low energy CMOS devices take the advantage of complimentary connected network of thousands of metal oxide synaptic devices.

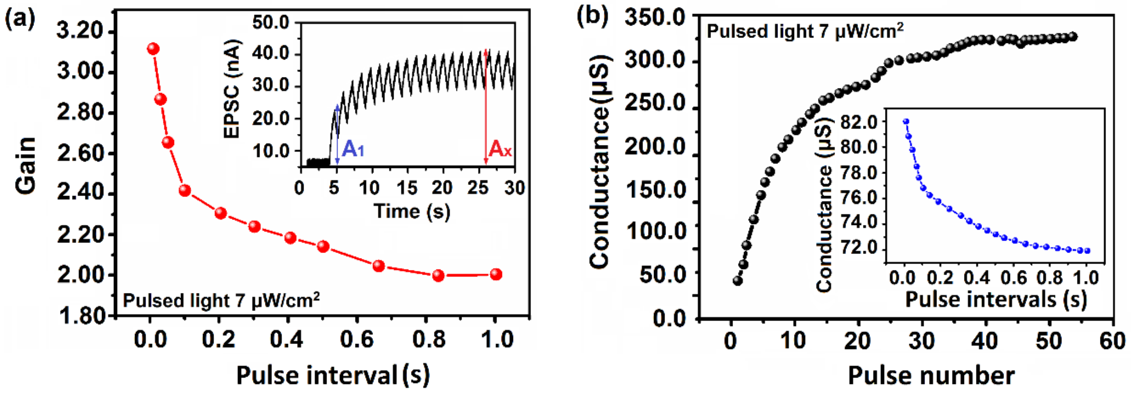

Supplementary Figure 11. (a) The impact of pulse intervals on the gain characteristics of devices. Gain is defined as the ratio of A_x/A_1 (Inset in Figure 11a). Here the twentieth peak was chosen as the representative of A_x. (b) The impact of pulse number and pulse interval on conductance of optical synapses.

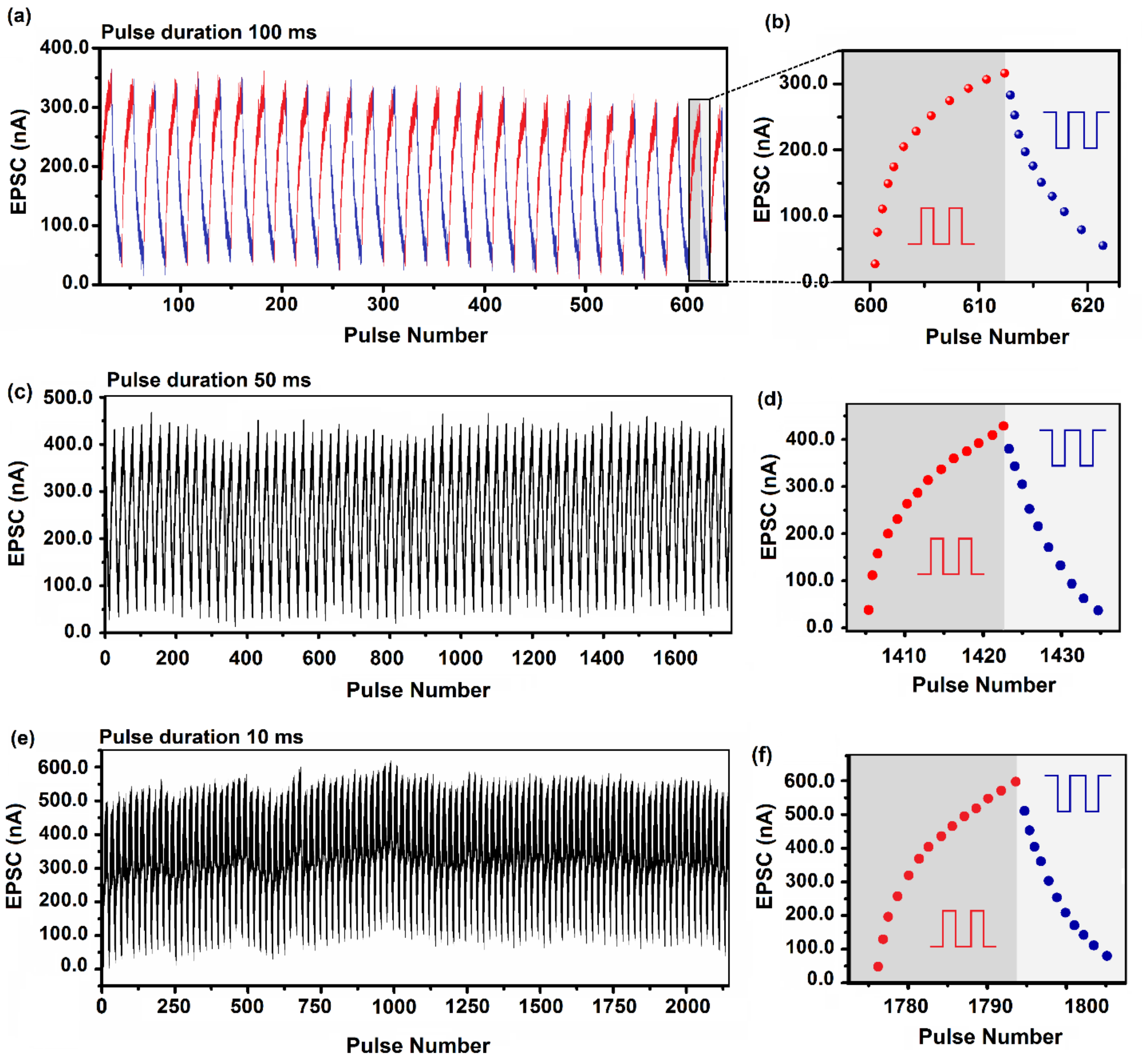

Supplementary Figure 12. Analog current modulation for sequentially repeated optical pulses (7 mW cm^-2), respectively with (a) 100 ms, (c) 50 ms and (e) 10 ms pulse durations, followed by consecutive -10 mV depression voltage pulses. The increase and decrease of EPSC is the manifestation of LTP and LTD in a biological system[18].

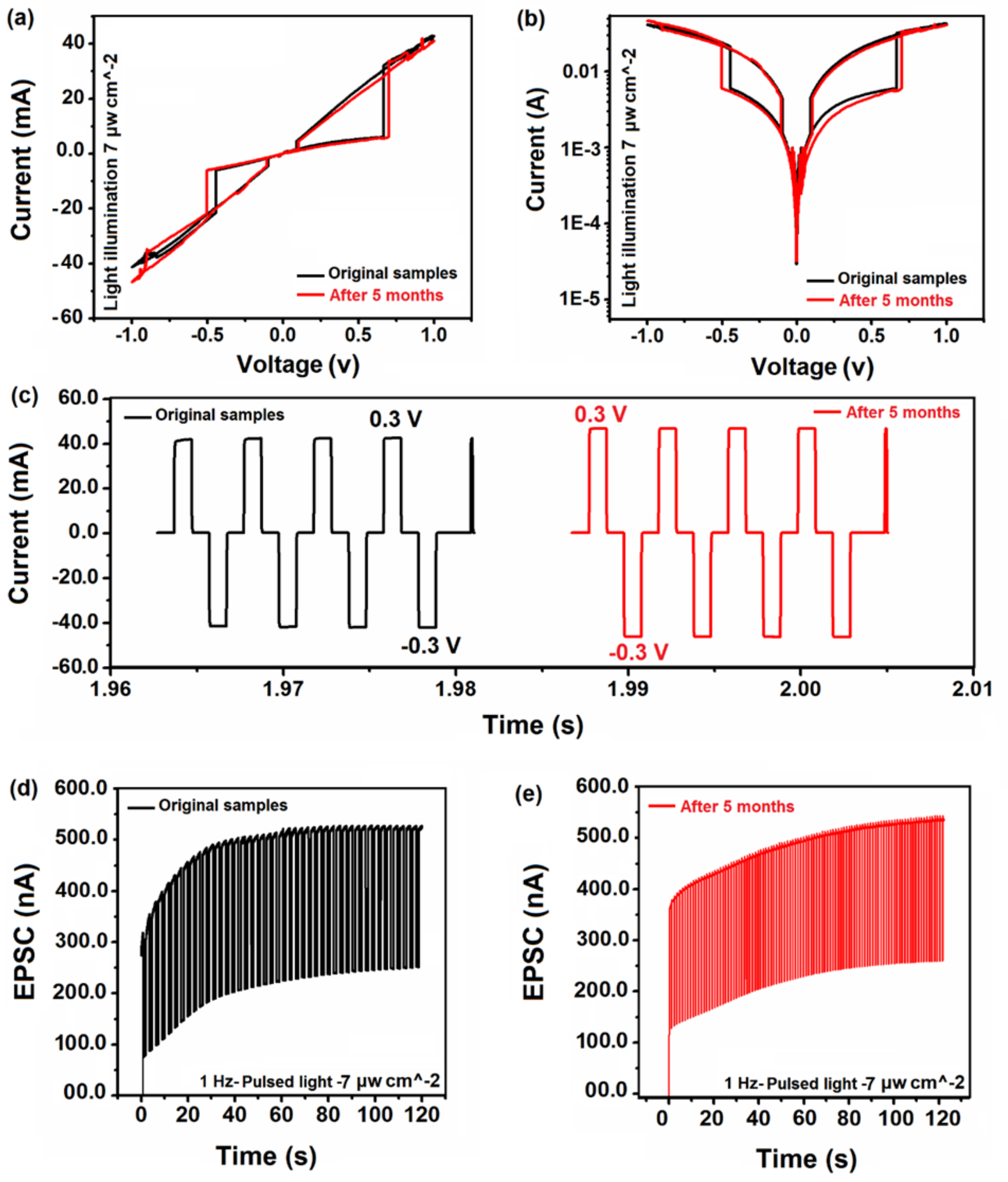

Supplementary Figure 13. The device-to-device uniformity of synapses. (a) The potentiodynamic (*I*-*V*) curves of ITO/In-doped TiO_2/Au device and (b) its corresponding logarithmic scale of graph. (c) The pulse driven element operation of original Pt/In-doped TiO_2/Au devices and another similar device after 5 months. (d) The EPSC graph of original ITO/In-doped TiO_2/Au optical synaptic device for sequential light pulses with 1HZ frequency and (e) the same measurements after 5 months for the another similar device.

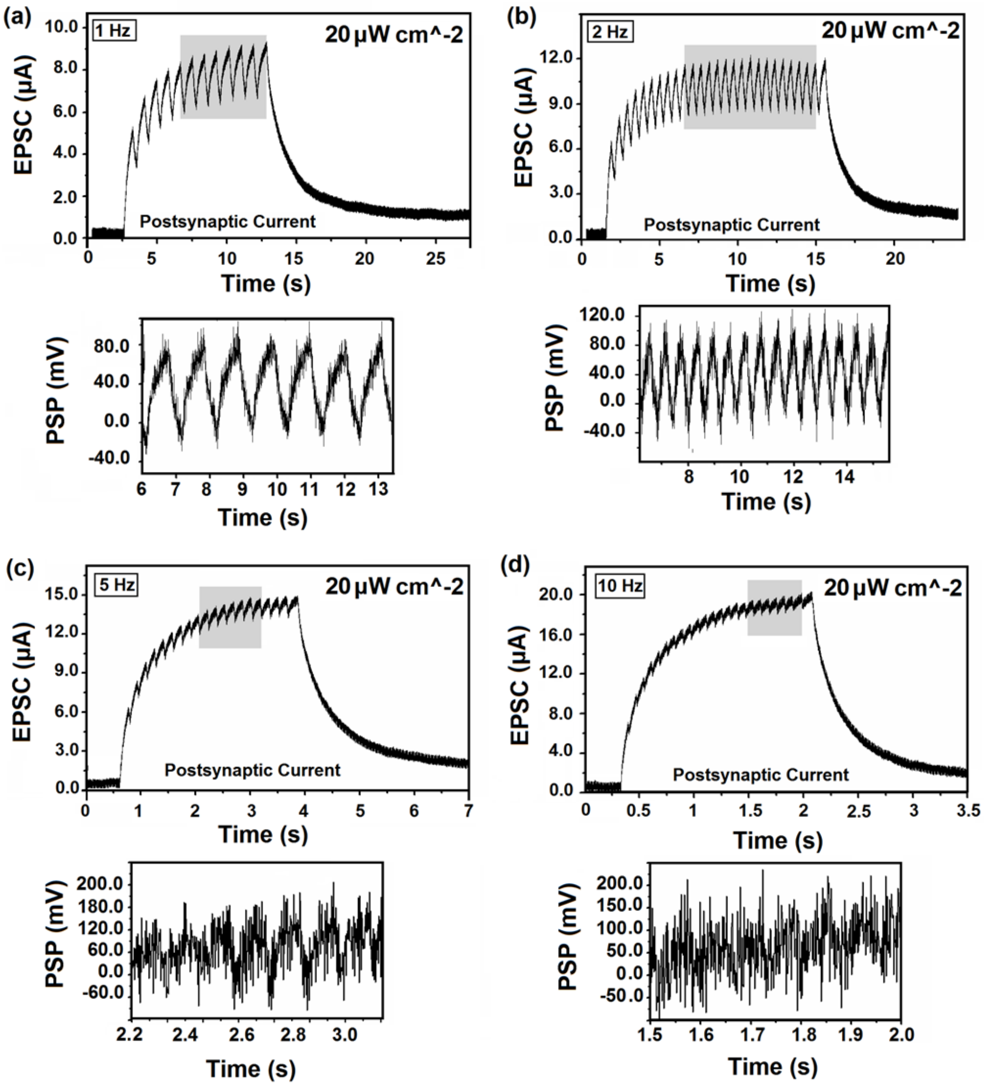

Supplementary Figure 14. The effect of frequency of pulsed light (20 μW cm^-2) on EPSC and PSP of ITO/In-doped TiO_2/Au synaptic device. (a) 1 Hz, (b) 2Hz, (c) 5 Hz, (d) 10 Hz.

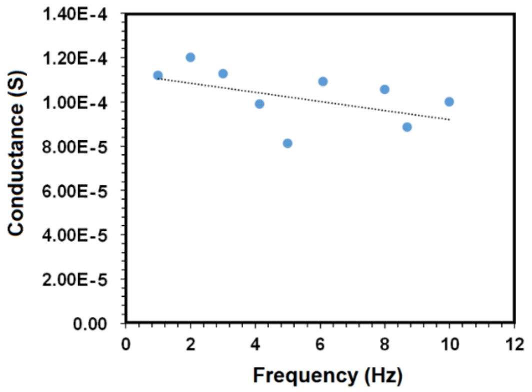

Supplementary Figure 15. The variation of conductance vs. the frequency of pulsed lights.

Supplementary Note 9. Lippmann equation

When the voltage drops across the EDL, the surface tension between the electrolyte and liquid metal can be calculated by using the Lippmann equation[19]:

$$\gamma = \gamma_0 - \frac{1}{2}\, cV^2 \qquad (1)$$

where, γ is the surface tension, γ_0 is the maximum of surface tension when applied potential is 0, c is the capacitance of EDL per unite area and V is the potential difference across the EDL. When the applied voltage is 0, the EDL is charged by uniformly distributed q_0 over the surface of galinstan droplet. In this condition the potential difference across the EDL is defined by $V_0 = \frac{q_0}{c}$.

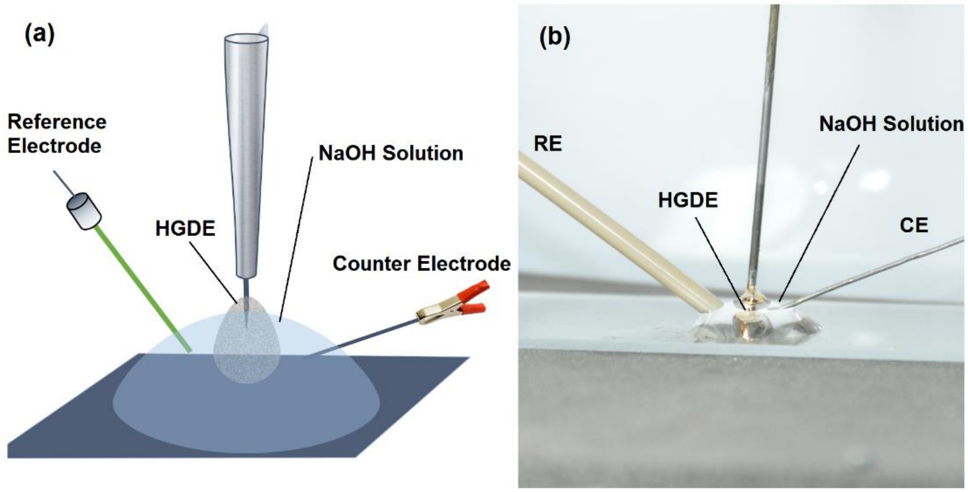

Supplementary Figure 16. (a) The schematic representation of hanging galinstan drop electrode (HGDE) and (b) the optical image of its actual set up.

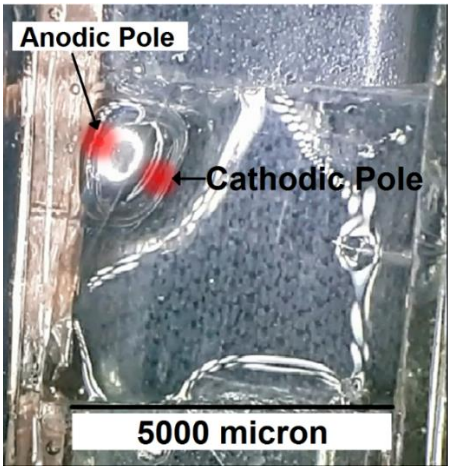

Supplementary Figure 17. The schematic depiction of in-situ Raman studies of anodic and cathodic poles. To this aim, continues Raman mode is employed. The laser beam is focused on the specific conical part of droplet which is semi-submersed into electrolyte.

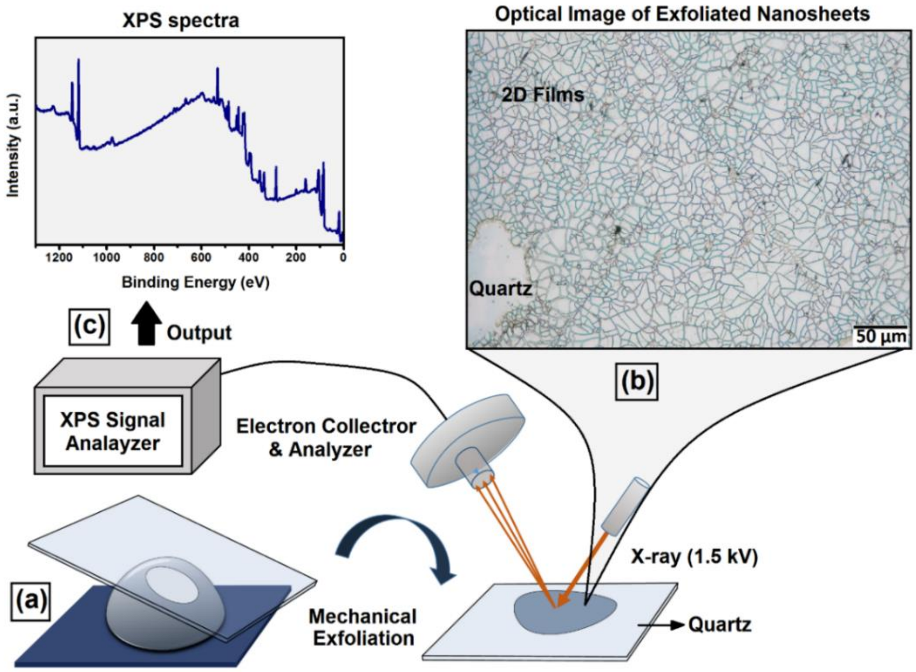

Supplementary Figure 18. (a) The scheme of mechanical exfoliation of surface oxide of galinstan droplet, (b) optical image of exfoliated nanosheets and (c) the XPS results of nanosheets.

Supplementary Note 10. Details of image analysis:

To determine the strain values during oscillation, images were taken by a high speed camera and then analyzed frame by frame using image analyzer to determine the area of droplets during mechanical strain. A polygon mesh model of the surface was developed based on the generated 3D image (***iSolution Lite X64***) of the surface. Considering the scale bar of figures, the surface area of droplet was estimated by counting the number of squares over the surface which has different optical contrasts with the surrounding area. To be precise, several images with different contrasts and background colors were produced. A typical developed images are shown in Figure 19.

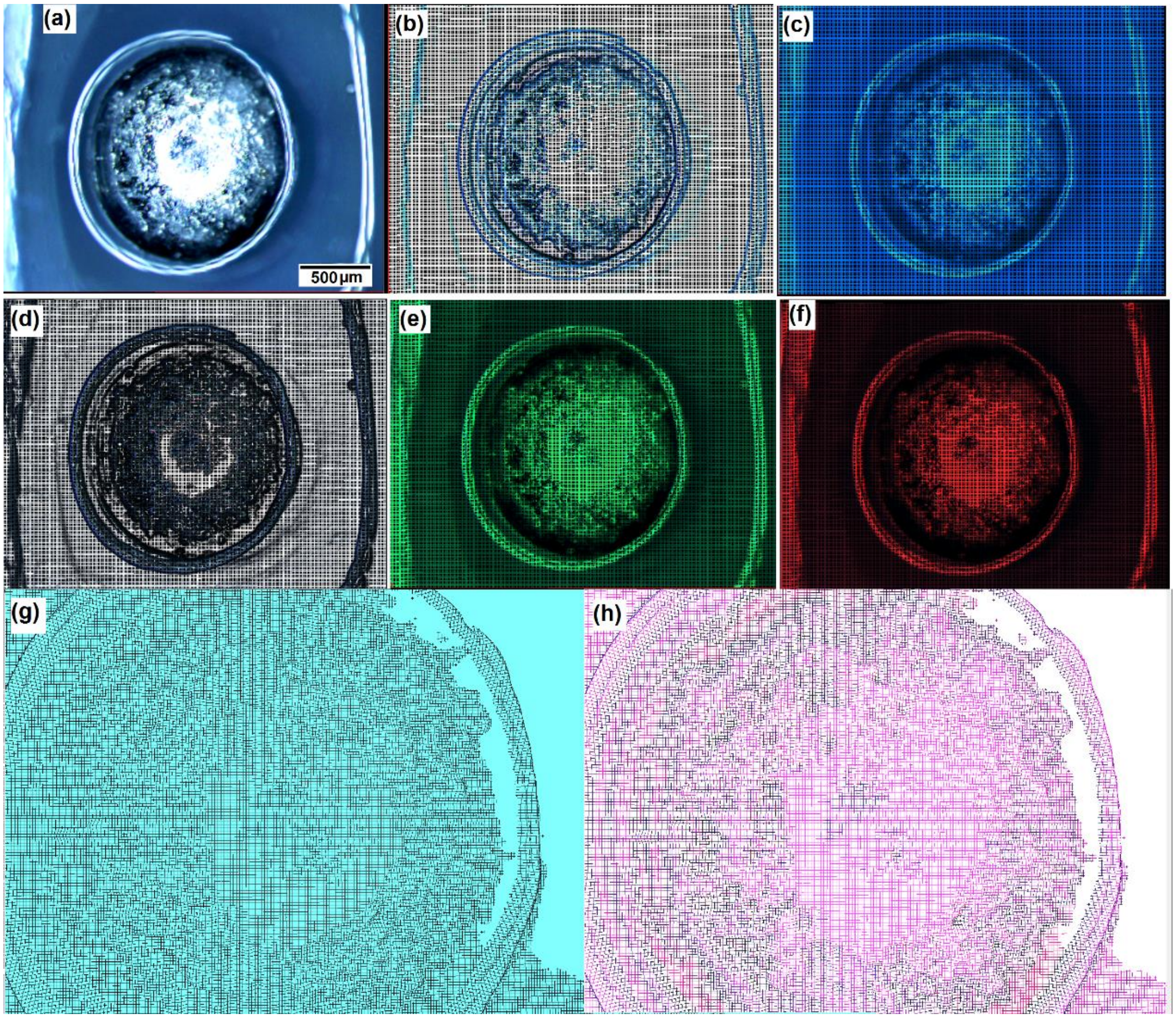

Supplementary Figure 19. Automated analysis. (a) The actual image of galinstan droplet and (b~h) the typical polygon mesh structures developed from 3D models.

References:

1. Yang, C. S. et al. All-solid state synaptic transistor with ultralow conductance for neuromorphic computing. *Adv. Funct. Mater.* 1804170 (2018).
2. Wedig, A. et al. Nanoscale cation motion in TaO_x, HfO_x and TiO_2 memristive systems. *Nat. Nanotechnol.* **11**, 67–74 (2016).
3. Xu, W., Min, S. Y., Hwang, H. & Lee. T. W. Organic core-sheath nanowire artificial synapses with femtojoule energy consumption. *Sci. Adv.* **2**, e1501326 (2016).
4. Zhu, L. Q., Wan, C. J., Guo L. Q., Shi, Y., & Wan, Q. Artificial synapse network on inorganic proton conductor for neuromorphic systems. *Nat. Commun.* **5,** 3158 (2014).
5. Balakrishna, P. & De Souza, P. M. M. Nanoionics-Based three-terminal synaptic device using zinc oxide. *ACS Appl. Mater. Interfaces*, **2,** 1609–1618 (2017).
6. Yang, C. S. et al. A synaptic transistor based on quasi-2D molybdenum oxide. *Adv. Mater.* **29**, 1700906. (2017).
7. Jiang, L., Lv, F. C., Yang, R., Hu, D. C. & Guo, X. Forming-free artificial synapses with Ag point contacts at interface. *J. Materiomics.* (2018) https://doi.org/10.1016/j.jmat.2018.11.001.
8. Nawrocki, R. A., Voyles, R. M. & Shaheen, S. E. A mini review of neuromorphic architectures and implementations. *IEEE Trans. Electron Devices* **63,** 3819-3829 (2016).
9. Yu, S., Wu, Y., Jeyasingh, R., Kuzum, D. & Wong, H. P. An electronic synapse device based on metal oxide resistive switching memory for neuromorphic computation *IEEE Trans. Electron Devices* **58,** 2729- 2737 (2011).
10. Yu, S., Gao, B., Fang, Z., Yu, H., Kang, J. & Wong, H. S. P. A low energy oxide-based electronic synaptic device for neuromorphic visual systems with tolerance to device variation. *Adv. Mater.* **25**, 1774–1779 (2013).
11. Kuzum, D., Yu, S. & Wong, H. S. P. Synaptic electronics: materials, device and applications. *Nanotechnology* **24,** 382001 (2013).
12. Yang, R. et al. On-demand nanodevice with electrical and neuromorphic multifunction realized by local ion migration. *ACS Nano* **6**, 9515-9521 (2012).
13. Park, S, et al. RRAM-based synapse for neuromorphic system with pattern recognition function. 2012 IEEE Int. Electron Devices Meeting (IEDM).
14. Yu, S., Gao, B., Fang, Z., Yu, H., Kang, J. & Wong, H. S. P. Stochastic learning in oxide binary synaptic device for neuromorphic computing. *Front. Neurosci.* 7:186. (2013) https://doi.org/10.3389/fnins.2013.00186.
15. Yu, S., Gao, B., Fang, Z., Yu, H., Kang, J. & Wong, H. S. P. A neuromorphic visual system using RRAM synaptic devices with Sub-pJ energy and tolerance to variability: Experimental characterization and large-scale modeling. 2012 International Electron Devices Meeting, https://doi.org/10.1109/IEDM.2012.6479018.
16. Kumar, M., Abbas, S. & Kim, J. All-oxide based highly transparent photonic synapse for neuromorphic computing. *ACS. Appl. Mater. Interfaces.* **10**, 34370-34376 (2018).
17. Wang, I. T., Lin, Y. C., Wang, Y. F., Hsu, C. W., & Hou, T. H. 3D synaptic architecture with ultralow sub 10 fJ energy per spike for neuromorphic computation. 2014 IEEE International Electron Devices Meeting. https://doi.org/10.1109/IEDM.2014.7047127.
18. Van de Burgt, Y. et al. A non-volatile organic electrochemical device as a low-voltage artificial synapse for neuromorphic computing. *Nat. Mater.* **16**, 414-418 (2017).
19. Huttenloch, P., Roehl, K. E. & Czurda, K. Use of copper shavings to remove mercury from contaminated Groundwater or wastewater by amalgamation. *Environ. Sci. Technol.* **37**, 4269-4273 (2003).
